# Matching of O_2_ Utilization and O_2_ Delivery in Contracting Skeletal Muscle in Health, Aging, and Heart Failure

**DOI:** 10.3389/fphys.2022.898395

**Published:** 2022-06-14

**Authors:** Michael Nyberg, Andrew M. Jones

**Affiliations:** ^1^ Vascular Biology, Global Drug Discovery, Novo Nordisk A/S, Maaloev, Denmark; ^2^ Department of Sport and Health Sciences, University of Exeter, Exeter, United Kingdom

**Keywords:** O_2_ uptake kinetics, handgrip exercise, knee-extensor exercise, cycling, blood flow

## Abstract

Skeletal muscle is one of the most dynamic metabolic organs as evidenced by increases in metabolic rate of >150-fold from rest to maximal contractile activity. Because of limited intracellular stores of ATP, activation of metabolic pathways is required to maintain the necessary rates of ATP re-synthesis during sustained contractions. During the very early phase, phosphocreatine hydrolysis and anaerobic glycolysis prevails but as activity extends beyond ∼1 min, oxidative phosphorylation becomes the major ATP-generating pathway. Oxidative metabolism of macronutrients is highly dependent on the cardiovascular system to deliver O_2_ to the contracting muscle fibres, which is ensured through a tight coupling between skeletal muscle O_2_ utilization and O_2_ delivery. However, to what extent O_2_ delivery is ideal in terms of enabling optimal metabolic and contractile function is context-dependent and determined by a complex interaction of several regulatory systems. The first part of the review focuses on local and systemic mechanisms involved in the regulation of O_2_ delivery and how integration of these influences the matching of skeletal muscle O_2_ demand and O_2_ delivery. In the second part, alterations in cardiovascular function and structure associated with aging and heart failure, and how these impact metabolic and contractile function, will be addressed. Where applicable, the potential of exercise training to offset/reverse age- and disease-related cardiovascular declines will be highlighted in the context of skeletal muscle metabolic function. The review focuses on human data but also covers animal observations.

## Introduction

Skeletal muscle is one of the most dynamic metabolic organs. Metabolic rate can increase from values at rest of ∼0.02 up to ∼3.7 mmol ATP kg^−1^ s^−1^ during maximal intensity exercise (Hargreaves and Spriet, 2020). Given the relatively small intracellular stores of ATP (∼5 mmol kg^−1^), activation of metabolic pathways is required to maintain the necessary rates of ATP resynthesis during sustained contractile activity. These pathways include phosphocreatine and glycogen breakdown that allows for substrate-level phosphorylation (anaerobic) and oxidative phosphorylation (aerobic). The latter becomes increasingly important as the duration of activity increases and oxidative phosphorylation is already the major ATP-generating pathway when contractile activity extends beyond ∼1 min.

Oxidative metabolism of carbohydrate and fat is highly dependent on the ability of the cardiovascular system to deliver O_2_ to the contracting muscle fibres. In skeletal muscle, convective O_2_ delivery, which refers to the active movement of O_2_ in blood, determines the rates of O_2_ that enters the muscle. At the microcirculatory level, O_2_ diffuses across the capillary wall, interstitium and sarcolemma (diffusive O_2_ transport). Hence, the number (total surface area) and spatial distribution of capillaries within the muscle, rate of red blood cell entry and red cell velocity in each capillary, and O_2_ saturation are important factors for O_2_ transfer to, and utilization by, mitochondria (Egginton and Gaffney, 2010; Mendelson et al., 2021).

The increase in blood flow to contracting skeletal muscle is achieved through local vasodilation that is the result of a complex interplay between vasoconstrictor and vasodilator signalling (Joyner and Casey, 2015). Reports on maximal skeletal muscle blood flow during exercise in different species including humans have demonstrated values in the range of 2–4 L kg tissue^−1^ depending on species, fibre type composition, and training status (Armstrong and Laughlin, 1983; Andersen and Saltin, 1985; Richardson et al., 1995; Mortensen et al., 2008; Joyner and Casey, 2015). As skeletal muscle comprises ∼30%–40% of the total body mass, intense exercise engaging a large proportion of the total muscle mass will pose a threat to arterial blood pressure as maximal cardiac output is insufficient to offset the reduction in peripheral vascular resistance (Calbet et al., 2004; Rowell, 2004). Consequently, a reduction in perfusion and O_2_ delivery to the contracting muscles obtained through sympathetic constraint of vasodilation is needed to maintain arterial blood pressure and ensure sufficient perfusion of vital organs such as the brain (Rowell, 2004; Joyner and Casey, 2015).

Advancing age is associated with a broad range of alterations in the cardiovascular system such as augmented arterial stiffness that increases afterload on the left ventricle, systolic blood pressure, left ventricular mass, and reduced left ventricular diastolic performance (Lakatta and Levy, 2003b, a; Fleg and Strait, 2012). At the level of skeletal muscle, declines in skeletal muscle mass, endothelial and mitochondrial function, and reduced capillarization and blood flow to exercising muscle are all hallmarks of aging (Nyberg and Hellsten, 2016). These changes in the cardiovascular and skeletal muscle systems are, however, not unique for the aging process, as they are also manifested in a broad range of cardiovascular diseases such as different forms of heart failure. Importantly, many biological alterations that come with advancing age and cardiovascular diseases are the result of a complex interplay between systems that are influenced by genetic and life-style factors. Regarding the latter, one important factor is physical activity due to its profound effects on cardiovascular structure and function, and physical inactivity induces many of the cardiovascular changes associated with aging (Saltin et al., 1968; McGuire et al., 2001a, b).

The present review is composed of two parts. In the first part, local and systemic mechanisms involved in the regulation of O_2_ delivery and how integration of these influences matching of skeletal muscle O_2_ demand and O_2_ delivery will be addressed. Here, the discussions will be centred around exercise engaging a small muscle mass in the form of handgrip and knee-extensor exercise, and those engaging larger proportions of the total mass such as cycling, running, rowing, and cross-country skiing. It should be noted that the physiological responses discussed in this first section represents that of younger (∼18–35 years) subjects in which no significant age-related alterations in cardiovascular function are expected. Furthermore, since most of the studied subjects have been males, one should be cautious when interpreting and generalising the findings. In the second part, the focus will be on how aging and heart failure lead to in part common alterations in cardiovascular structure and function, and how these impact metabolic and contractile function of skeletal muscle. In addition, the potential of exercise training to offset/reverse age- and disease-related cardiovascular declines will be highlighted in the context of skeletal muscle metabolic function.

## Matching of Skeletal Muscle O_2_ Demand and Delivery

**FIGURE 1:**
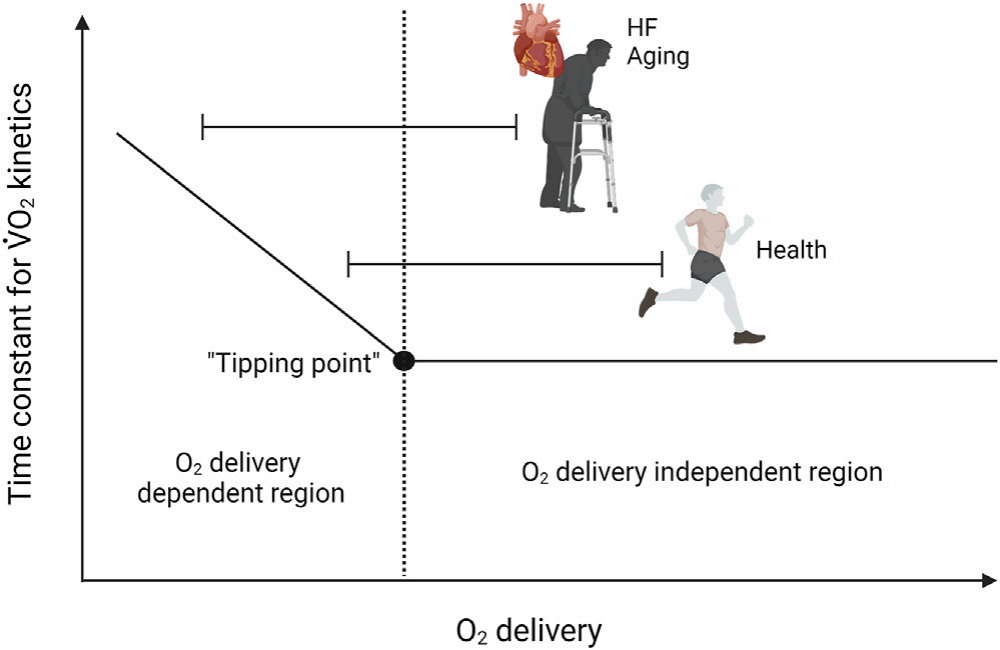
Model demonstrating the effects of altering the rate of O_2_ delivery on skeletal muscle O_2_ utilization. If an increase or decrease in the rate of O_2_ delivery does not lead to a change in the rate of oxidative metabolism, this suggests that O_2_ availability in the control condition is in excess of demand (flat portion’ of the relationship between the rate of O_2_ utilization and the rate of O_2_ delivery). Conversely, if an increase in the rate of O_2_ delivery enhances the rate of O_2_ consumption or speeds O_2_ uptake kinetics following the onset of exercise, mitochondrial oxidative phosphorylation is likely to be limited by O_2_ availability. In a situation where a reduction in O_2_ delivery reduces the rate of O_2_ utilisation or slows O_2_ uptake kinetics following the onset of exercise, O_2_ supply in the control state would not necessarily be inadequate but could be very closely matched to O_2_ demand (right on the tipping point). Note that aging and HF shifts the operating domain to the left on the continuum.

The matching of skeletal muscle O_2_ utilization through mitochondrial oxidative phosphorylation and O_2_ delivery can be assessed through experiments in which O_2_ delivery is manipulated and can be understood with reference to the “tipping point” hypothesis of Poole et al. (2008) (Figure 1). In such experiments, if an increase or decrease in the rate of O_2_ delivery does not lead to a change in the rate of oxidative metabolism, this suggests that O_2_ availability in the control condition is in excess of demand, i.e., that the control condition is situated on the “flat portion” of the relationship between the rate of O_2_ utilization and the rate of O_2_ delivery. Conversely, if an increase in the rate of O_2_ delivery enhances the rate of O_2_ consumption or speeds O_2_ uptake kinetics following the onset of exercise, mitochondrial oxidative phosphorylation is likely to be limited by O_2_ availability. In a setting where a reduction in O_2_ delivery reduces the rate of O_2_ utilisation or slows O_2_ uptake kinetics following the onset of exercise, O_2_ supply in the control state would not necessarily be inadequate but could be considered to be very closely matched to O_2_ demand, i.e., to be situated “right on” the tipping point. In addition to the rate of O_2_ consumption, changes in the rate of substrate-level phosphorylation (evidenced, for example, by changes in muscle phosphocreatine and lactate concentrations), force output, and the ability to perform a given task will also provide essential insight into the extent to which oxidative metabolism is affected by manipulation of O_2_ delivery.

There are several ways of experimentally increasing perfusion of skeletal muscle. By arterial infusion of a vasodilator substance and by increasing perfusion pressure through changes in hydrostatic pressure (e.g., arm positioned below *vs.* above the level of the heart), blood flow and O_2_ delivery to the contracting muscle can be enhanced. Notably, although breathing hyperoxic air increases blood O_2_ content, this does typically not increase the rate of O_2_ delivery due to reduction in blood flow to maintain the rate of O_2_ delivery (Gonzalez-Alonso et al., 2002). Reductions in skeletal muscle O_2_ delivery can be obtained by arterial infusion of a vasoconstrictor substance and by increasing sympathetic nervous activity and constraint of vasodilation. By introducing hypoxia, large reductions in arterial O_2_ content can be achieved. As also discussed later, a compensatory increase in blood flow that allows O_2_ delivery to be maintained is observed in some circumstances. In the following sections, various models in which the O_2_ delivery has been experimentally altered will be used to provide insight into how skeletal muscle O_2_ demand and delivery are matched.

### Exercise Engaging a Small Muscle Mass

#### Knee-Extensor Exercise

The initial phase of exercise is characterized by a rapid increase in O_2_ delivery to meet the higher metabolic demand of the contracting muscle. During moderate-intensity knee-extensor exercise, which engages 2–3 kg of muscle mass, the increase in O_2_ delivery in the initial phase appears to supersede oxidative metabolism. This may in part relate to mechanical factors, as deformation obtained *via* high extravascular pressure (Kirby et al., 2007) and passive limb movement (Mortensen et al., 2012a) have been shown to elicit rapid vasodilation that decreases over time. This mechanically induced vasodilation could serve as a feed-forward mechanism during the very early phase of exercise where metabolic vasodilation is less prominent. Such a mechanism is in line with the observation that the difference between skeletal muscle O_2_ delivery and O_2_ uptake is greatest in the initial phase of exercise (Nyberg et al., 2010), although it is important to underscore that this relationship may also be reflective of a potential hyperperfusion in areas of the muscle that are inactive as well as in non-recruited muscles. Nevertheless, a perfusion limitation appears to be unlikely as a reduction in exercise-induced leg blood flow and O_2_ delivery by ∼25%–45%, obtained through pharmacological inhibition of nitric oxide synthase (NOS) and cyclooxygenase (COX), does not alter skeletal muscle O_2_ uptake due to compensatory increase in O_2_ extraction (Nyberg et al., 2010). It should be noted that the contraction-induced change in blood flow in this study did not appear to be affected by the pharmacological blockade (as the difference was present during passive movement of the leg) and future studies should address how significant alterations in blood flow kinetics may affect the rate of O_2_ consumption.

As opposed to moderate-intensity exercise where predominantly slow-twitch (ST) fibres are engaged, both ST and fast-twitch (FT) fibres are recruited during more intense exercise (Krustrup et al., 2004). In humans, FT fibres have a lower oxidative capacity than ST fibres (Essen-Gustavsson and Henriksson, 1984) and the rise in skeletal muscle O_2_ uptake is also slowed in the transition from moderate to intense knee-extensor exercise (Nyberg et al., 2021). Additionally, O_2_ delivery relative to leg O_2_ utilization appears to be reduced in an intensity-dependent manner (Nyberg et al., 2010; Christensen et al., 2013; Nyberg et al., 2014). Hence, a “tipping point” regarding O_2_ delivery beyond which O_2_ uptake kinetics become progressively slowed with further reductions in O_2_ delivery may exist during intense exercise (Poole et al., 2008). Similar to findings during moderate-intensity knee-extensor exercise (Nyberg et al., 2010), pharmacological inhibition of NOS and COX reduced exercise-induced skeletal muscle O_2_ delivery by ∼25%–50% in the initial phase of intense knee-extensor exercise (∼86% of incremental test peak power) (Christensen et al., 2013). In this setting, however, the rise in skeletal muscle O_2_ uptake was slowed despite a higher O_2_ extraction. This finding suggests that FT fibres are more sensitive to a reduction in O_2_ delivery than ST fibres but also emphasizes that a marked reduction in O_2_ availability cannot be tolerated at higher intensities. To gain more clarity on how close to the tipping point the muscle was operating, experiments in which O_2_ delivery are reduced to a lesser extent are warranted. Notably, increasing blood flow and O_2_ delivery ∼2-fold by arterial ATP infusion in a similar bout of intense knee-extensor exercise does not increase skeletal muscle O_2_ uptake in the initial phase of exercise (Nyberg et al., 2014), suggesting that O_2_ availability still matches or exceeds mitochondrial capacity for oxidative phosphorylation in the control situation.

During continuous knee-extensor exercise, skeletal muscle blood flow and O_2_ delivery is closely matched to the rate of O_2_ consumption across the full intensity spectrum (Andersen and Saltin, 1985; Mortensen et al., 2008). This matching, which is the result of a complex interplay between the sympathetic nervous system and local vasoactive systems (Hellsten and Nyberg, 2015; Joyner and Casey, 2015), is important to secure adequate O_2_ availability in the contracting fibres. One example of this is in hypoxic conditions where reductions in arterial O_2_ content by up to ∼25% is compensated by an increase in blood flow that allows O_2_ delivery and utilization to be maintained at moderate intensities (Koskolou et al., 1997; Gonzalez-Alonso et al., 2002; Mortensen et al., 2011). The importance of this matching between O_2_ supply and utilization is further underscored by the great degree of redundancy that exists between vasodilator systems regulating skeletal muscle blood flow that allows for preservation of blood flow in conditions where one or more vasoactive systems may be compromised (Hellsten and Nyberg, 2015; Joyner and Casey, 2015). At moderate intensity, there even appears to be hyperperfusion of the exercising knee-extensors as a pharmacologically induced reduction in leg blood flow and O_2_ delivery of ∼10%–25% is compensated by increased O_2_ extraction so that skeletal muscle O_2_ utilization is maintained (Nyberg et al., 2010; Mortensen et al., 2012b; Nyberg et al., 2015a).

During sustained intense knee-extensor exercise, skeletal muscle perfusion appears to more closely match O_2_ demand. Combined inhibition of NOS and COX, which reduces exercise-induced blood flow and O_2_ delivery by ∼20% during moderate-intensity exercise (Nyberg et al., 2010), did not reduce O_2_ delivery and utilization despite pronounced effects on these variables in the initial phase of exercise (Christensen et al., 2013). The mechanism underlying the preservation of blood flow is likely to involve compensatory contribution from redundant vasodilator systems that were activated, which may have been a direct effect of insufficient skeletal muscle O_2_ availability. Interestingly, when blood flow was increased through arterial ATP infusion during intense exercise, O_2_ uptake was found to be reduced after 30 s and until predetermined exercise termination at 4 min (Nyberg et al., 2014). This observation may relate to the capacity of intravascular ATP to override sympathetic vasoconstrictor activity (Rosenmeier et al., 2004; Mortensen et al., 2012b). During exercise, sympathetic activity reduces perfusion of inactive muscles whereas this effect is blunted in contracting muscle (termed functional sympatholysis), thus directing blood flow away from areas of lower metabolic activity and toward areas of higher metabolic demand (Remensnyder et al., 1962; Saltin and Mortensen, 2012). Importantly, the vasoconstrictor effects of muscle sympathetic nervous activity are not abolished (Remensnyder et al., 1962; Buckwalter et al., 2001), and sympathetic restraint of blood flow remains even in highly active skeletal muscle (Joyner et al., 1992; Buckwalter and Clifford, 2001). In the setting of ATP-induced vasodilation in regions under sympathetic vasoconstrictor control, the precise matching of O_2_ delivery and demand at the microvascular level may be disturbed as selective and controlled perfusion of vessels supplying regions in need of O_2_ is required for optimal tissue oxygenation (Sprague et al., 2010; Ellsworth et al., 2016). Given the very high blood flow rates (∼50% above control), mean transit time in capillaries perfusing highly active fibres may also have reached levels that do not allow for sufficient O_2_ off-loading from haemoglobin.

Taken collectively, the above findings highlight the close matching that exist between skeletal muscle O_2_ delivery and oxidative metabolism and indicate that the knee-extensor muscles may be operating closer to a tipping point with regards to O_2_ delivery with increasing exercise intensity; however, the regulation of O_2_ consumption still resides at the level of the mitochondria.

#### Handgrip Exercise

Hand gripping is evidently a small muscle mass exercise as <1 kg of muscle is being activated. However, as compared to the locomotor muscles of the lower extremities, the muscles in the forearm serve different functions such as ensuring very precise and coordinated movements. Furthermore, forearm muscles are subjected to lower hydrostatic pressures relative to the legs and they also display lower arterial wall thickness (Astrand et al., 2003), reduced adrenergic responsiveness (Pawelczyk and Levine, 2002; Nyberg et al., 2018), and enhanced response to vasodilator substances (Proctor and Newcomer, 2006). These structural and functional differences could entail differences in the matching of skeletal muscle O_2_ delivery and O_2_ demand. In accordance with this proposition, exercising with the arm below compared to above the level of the heart to augment perfusion pressure, is associated with increased blood flow and skeletal muscle O_2_ uptake in the initial phase of moderate-intensity exercise (Hughson et al., 1996). In a more recent study using a combination of diffuse correlation spectroscopy and near-infrared spectroscopy (NIRS), exercising with the arm above the heart was associated with slower blood flow kinetics, however, the observed ∼25% slower O_2_ uptake kinetics did not reach statistical significance (Tucker et al., 2019). By using a different experimental setup, skeletal muscle blood flow and O_2_ uptake was found to rise more rapidly when mean arterial blood pressure (MAP) was increased through stimulation of chemoreflexes in the calf muscles (Perrey et al., 2001). Furthermore, in a series of experiments in which prior exercise and/or ischemia were applied prior to intense exercise, a strong correlation between the rates of increase in blood flow and oxidative metabolism were observed (Faisal et al., 2010), suggesting that the acceleration of the skeletal muscle O_2_ uptake at the onset of intense forearm exercise is linked to O_2_ delivery.

In contrast to the apparent perfusion limitation across the intensity continuum at the onset of forearm exercise, some degree of surplus in O_2_ availability may exist during steady state conditions performed at moderate intensity. Evidence from this comes from steady state exercise, where a combination of hypoxia and pharmacological inhibition of NOS and COX, that led to a ∼10% reduction in O_2_ delivery, was associated with increased O_2_ extraction and preserved O_2_ uptake (Crecelius et al., 2011). Somewhat in line with this observation, a ∼30% increase in convective O_2_ delivery obtained through increased perfusion pressure was not associated with enhanced skeletal muscle O_2_ uptake at steady state, although a trend toward a higher value was detected (Tucker et al., 2019). It should also be noted that intra-arterial infusion of ATP has been shown to have an additive effect on exercise hyperaemia during moderate intensity exercise (Shepherd et al., 2016) and infusion of ATP at a rate sufficient to double resting blood flow, has no effect on the amount and rate of blood-debt repayment detected after exercise (Patterson and Shepherd, 1954). During more intense exercise, blood flow and O_2_ uptake appears to reach a plateau at ∼80% of maximal workload (Nyberg et al., 2017). This finding may indicate that O_2_ delivery is limiting oxidative metabolism during intense forearm exercise, but more evidence is needed to support this.

### Exercise Engaging a Large Muscle Mass

#### Role of Sympathetic Vasoconstriction in the Regulation of Blood Pressure and Blood Flow Redistribution

Skeletal muscle has substantial vasodilator capacity as evidenced by reports of maximal blood flow values of 3–4 L kg^−1^ min^−1^ in humans during isolated muscle contractions (Andersen and Saltin, 1985; Richardson et al., 1995). During exercise that engages a large muscle mass, this ability of skeletal muscle to vasodilate can potentially outstrip cardiac output, which will then pose a threat to mean arterial pressure that needs to be maintained at ∼100 mmHg to secure perfusion of vital organs such as the brain (Calbet et al., 2004). Based on this great vasodilator capacity, skeletal muscle has also been described as the “sleeping giant” (Rowell, 2004). In addition to increases in cardiac output, enhanced sympathetic activation elicits vasoconstriction in less active tissues and constraint of vasodilation in more active tissues to maintain total peripheral resistance and blood pressure (Buckwalter and Clifford, 2001; Hansen et al., 2020) and redirect blood flow from less metabolically active tissues to the exercising muscles (Perko et al., 1998). This was elegantly demonstrated in a study of cross-country skiers in which values for maximal vasodilation (vascular conductance) of legs and arms were obtained. In these subjects who are characterised by highly trained upper and lower extremities, maximal vasodilation of both arms and legs would lead to a drop in mean arterial pressure to ∼75 mmHg; however, pressure remained at ∼95 mmHg during maximal exercise due to peripheral constraint of vasodilation that reduced the need for cardiac output by ∼15% (Calbet et al., 2004).

In the kidney and liver, sympathetic vasoconstriction can reduce perfusion by ∼75% during intense exercise in humans, thus allowing for ∼10% of maximal cardiac output (∼2 L min^−1^) to be redistributed (Joyner and Casey, 2015). In active skeletal muscle, the magnitude of decrease in vascular conductance and blood flow is dependent on muscle sympathetic nervous activity, metabolic activity, and the ability of the muscle for functional sympatholysis with respiratory muscles being less affected than limb skeletal muscles (Remensnyder et al., 1962; Saltin, 2007; Saltin and Mortensen, 2012). Depending on the extent of vascular restraint, matching of skeletal muscle O_2_ delivery and demand may be altered to an extent that will affect metabolic performance as discussed in the following sections.

#### Cycling and Running Exercise

Cycling and running can be defined as large muscle mass exercise as these types of activity recruits ∼50% of the total mass. During cycling performed at submaximal intensities, evidence supports that the limitation to skeletal muscle oxidative metabolism resides at the level of the mitochondria both at onset of exercise as well as during steady state conditions. For instance, application of lower body negative pressure, which is known to lead to a significant reduction in leg blood flow during exercise, does not change pulmonary oxygen kinetics during moderate and heavy intensity cycling (Williamson et al., 1996). During steady state conditions, decreasing cardiac output and leg blood flow by use of β-adrenergic blockade lead to a compensatory increase in O_2_ extraction to maintain leg O_2_ uptake during cycling performed at ∼40%–84% of maximal O_2_ uptake (Pawelczyk et al., 1992).

During very intense maximal cycling exercise eliciting maximal O_2_ uptake, systemic and leg O_2_ delivery and utilization fail to sufficiently rise to meet the increase in metabolic demand, contributing to task failure (Gonzalez-Alonso and Calbet, 2003; Mortensen et al., 2005; Mortensen et al., 2008; Trinity et al., 2012). The blunting of cardiac output is associated with the attainment of maximal heart rate and a plateau or even a decrease in stroke volume (Gonzalez-Alonso and Calbet, 2003; Mortensen et al., 2005; Mortensen et al., 2008; Trinity et al., 2012) that is likely to reflect restrictions in ventricular filling (Munch et al., 2014). This central limitation changes the site of regulation of skeletal muscle O_2_ consumption from mitochondrial respiration to convective O_2_ delivery (Levine, 2008; Skattebo et al., 2020). This shift is supported by very low femoral venous O_2_ saturation levels that range from ∼15% in habitually active (Mortensen et al., 2005; Mortensen et al., 2008) and down to 8% in well-trained individuals (Gonzalez-Alonso and Calbet, 2003; Munch et al., 2014) during maximal intensity cycling. Of note, the remaining O_2_ in the venous drainage is likely to reflect perfusion of less active tissues such as the skin and bone as well as a diffusion limitation across the capillary wall, interstitium, and sarcolemma given the expected low capillary PO_2_ in regions of very high metabolic activity (Skattebo et al., 2020). In line with this central limitation, *ex vivo* measurements have demonstrated a 2-fold higher mitochondrial capacity relative to maximal *in vivo* O_2_ uptake (Boushel and Saltin, 2013). Moreover, 3–8 weeks of endurance training leads to improvements in maximal O_2_ uptake that are driven primarily by increases in systemic O_2_ delivery (Montero et al., 2015). However, it should be emphasized that peripheral adaptations are also evident and these are needed as a response to the training-induced increases in cardiac output and skeletal muscle blood flow (Hellsten and Nyberg, 2015).

In summary, a tipping point for O_2_ delivery is apparent during very intense and maximal aerobic cycling with critical power/speed typically being at around 80%–90% of VO_2max_. Perfusion during lower intensities is of a magnitude that allows for compensatory O_2_ extraction in situations where blood flow is compromised.

#### Exercise Engaging Arms and Legs

A commonly used approach to study the effects of exercising with a large muscle mass on systemic and local hemodynamics is the combined arm and leg model. In a meta-analysis, blood flow to the leg was found to decline ∼10% when higher intensities of arm exercise was superimposed on leg exercise (Secher and Volianitis, 2006). In this setting of reduced O_2_ delivery, a compensatory increase in O_2_ extraction to maintain O_2_ uptake is a general finding (Savard et al., 1989; Richter et al., 1992; Richardson et al., 1995) although one finding of reduced O_2_ uptake has been reported (Secher et al., 1977). A decline in skeletal muscle perfusion is also apparent in the arms when a large muscle mass is recruited, as evidenced by reductions in blood flow to the arms during combined arm and leg exercise. However, in contrast to the legs, the increase in O_2_ extraction is insufficient to maintain oxidative metabolism in arm muscles (Volianitis and Secher, 2002; Volianitis et al., 2003; Volianitis et al., 2004).

The reduction in O_2_ utilization in the face of a decline in O_2_ delivery in the arms during combined exercise agrees with the previously discussed observations from studies utilizing handgrip exercise in which O_2_ supply appears to be limiting oxidative metabolism both at the onset of exercise as well as during intense sustained contractions. Furthermore, a training-induced increase in peak arm muscle O_2_ uptake during arm-cranking was also demonstrated to be an effect of increases in convective and diffuse O_2_ transport (Boushel et al., 2014). It should be noted that during maximal exercise in untrained individuals, O_2_ saturation in the venous drainage of the legs (femoral vein) reaches levels of ∼15% (Mortensen et al., 2005; Mortensen et al., 2008), whereas venous blood returning from the arms (subclavian vein) remains 40% saturated (Volianitis et al., 2004). In well-trained cross-country skiers performing maximal diagonal skiing involving both arms and legs, venous O_2_ saturation in the subclavian vein has been reported to be ∼14%, however, femoral venous O_2_ saturation levels were still significantly lower at ∼7% (Calbet et al., 2005). This lower capacity for O_2_ extraction in the arms is likely to reflect higher heterogeneity in blood flow distribution, shorter mean transit time, smaller diffusing area, and larger diffusing distance (Calbet et al., 2005).

**FIGURE 2:**
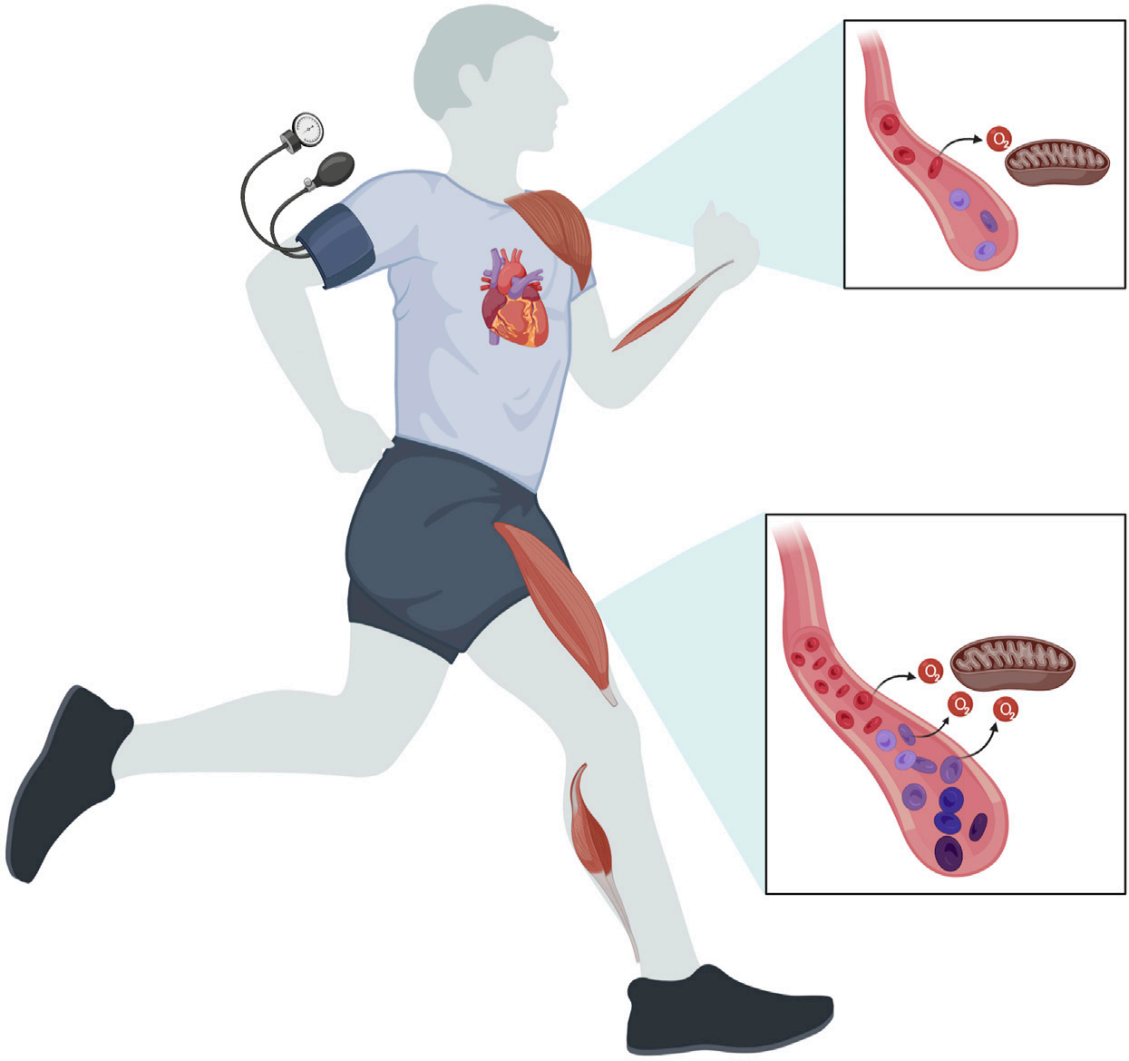
Regulation of skeletal muscle O_2_ delivery to match oxidative metabolism and maintain arterial blood pressure. During exercise engaging a small muscle mass such as handgrip and knee-extensor exercise, skeletal muscle blood flow and O_2_ delivery is closely matched to the metabolic demands of contraction. Here, the magnitude of perfusion allows for an increased O_2_ extraction so that skeletal muscle O_2_ utilization is maintained even in the setting of reduced blood flow, thus placing the limitation for oxidative metabolism at the level of the mitochondria. This hyperperfusion is most prominent in the lower extremities. During intense exercise engaging a large proportion of the total muscle mass, arterial pressure may be compromised as maximal cardiac output is insufficient to offset the reduction in peripheral vascular resistance. Consequently, vasoconstriction is needed in the active muscles to maintain arterial blood pressure to ensure sufficient perfusion of vital organs such as the brain, thus reducing perfusion and O_2_ delivery to the contracting muscles. In this setting, leg skeletal muscle hemodynamics and vascular network architecture allows for greater increases in O_2_ extraction compared to arm muscles. In both upper and lower extremities, the limitation to skeletal muscle oxidative metabolism during very intense/maximal exercise resides at the level of systemic and local convective O_2_ transport.

Taken collectively, the findings from the various types of exercise suggest that leg skeletal muscle hemodynamics and vascular network architecture allows for greater increases in O_2_ extraction without compromising skeletal muscle O_2_ consumption across the entire intensity and recruited muscle mass continuum (Figure 2). Given the locomotor function of the legs, it is not surprising that evolution would favour such a physiological system in which multiple redundant systems secure sufficient O_2_ delivery to muscles involved in movement.

## Effect of Aging and Cardiometabolic Diseases on the Matching of Skeletal Muscle O_2_ Demand and O_2_ Delivery

### Aging

Aging poses the largest risk factor for cardiovascular disease (North and Sinclair, 2012). Pathological alterations such as cardiac hypertrophy, altered left ventricular (LV) diastolic function, diminished LV systolic reverse capacity, increased arterial stiffness, and impaired endothelial function are all associated with the aging process (Lakatta and Levy, 2003b, a). In skeletal muscle, declines in muscle mass, force generation, endothelial and mitochondrial function, and capillarization are all hallmarks of aging and these alterations could potentially underlie the lower exercise-induced blood flow that has been reported in some studies (Kirby et al., 2009; Nyberg and Hellsten, 2016). Hence, both systemic and peripheral limitations may affect skeletal muscle metabolic performance in older individuals.

#### Handgrip Exercise

In older individuals, contraction-induced rapid-onset vasodilation as well as blood flow and vasodilator kinetics are slowed in forearm muscles (Casey and Joyner, 2012; Casey et al., 2015; Hughes et al., 2018). This blunted hemodynamic response has been proposed to be caused by blunted NO signaling (Casey et al., 2015). During sustained contractions, forearm vascular conductance and blood flow have similarly been described to be lower in aged individuals with more pronounced reductions at higher intensities (Jasperse et al., 1994; Kirby et al., 2012; Richards et al., 2014; Casey et al., 2015). This reduction in exercise hyperaemia have been reported to be related to less exercise-induced NO- and prostanoid-mediated vasodilatation, increased sympathetic outflow, and impaired functional sympatholysis (Taylor et al., 1992; Dinenno et al., 2005; Schrage et al., 2007). Despite the apparent alterations in skeletal muscle O_2_ delivery it remains unclear to what extent the rate of O_2_ consumption is affected as most studies have focused on regulation of vascular tone and blood flow. In one study, blood flow was found to be reduced with no compensatory increase in O_2_ extraction. This led to a lowering of skeletal muscle O_2_ utilisation by ∼7%–17% (at 5%–25% MVC), however, this did not reach statistical significance (Kirby et al., 2012). A potential effect on O_2_ uptake is supported by observations made with acute ascorbic acid supplementation that increased skeletal muscle blood flow and O_2_ uptake in older individuals (Richards et al., 2015). As previously discussed, forearm muscles operate close to the tipping point in terms of O_2_ delivery in healthy individuals. Hence, it is very plausible that age-related impairments in convective O_2_ transport could be limiting oxidative metabolism; however, more evidence is needed to support this.

#### Knee-Extensor Exercise

In the setting of knee-extensor exercise, older individuals display a slower increase in skeletal muscle vascular conductance, blood flow, and O_2_ delivery in the transition from rest to steady-state exercise at low- and moderate-intensity (Piil et al., 2018). Notably, the rate of increase in O_2_ uptake was similar in the group of young and older individuals because of higher a-vO_2_ difference in the older group. In the same group of older individuals, exercise training augmented the increase in vascular conductance and blood flow during the onset of moderate-intensity exercise without altering the rate O_2_ uptake. These initial observations suggest that skeletal muscle O_2_ delivery is not limiting for O_2_ utilisation in the initial phase of low- and moderate-intensity exercise engaging only the knee-extensors.

Skeletal muscle vascular conductance, blood flow, and O_2_ delivery have been reported to be lower in older individuals during steady state knee-extensor exercise, which may be a consequence of impaired endothelial function, functional sympatholysis, cGMP signalling and/or endothelin A mediated vasoconstriction (Donato et al., 2006; Mortensen et al., 2012b; Nyberg et al., 2012; Barrett-O'Keefe et al., 2015; Nyberg et al., 2015b). To what extent the attenuation in O_2_ delivery has consequences for the rate of O_2_ utilisation is currently unclear. In one group of older life-long sedentary individuals, skeletal muscle O_2_ delivery and O_2_ uptake were reported to be lower with no compensatory increase in a-vO_2_ difference (Mortensen et al., 2012b; Nyberg et al., 2012), thus indicating impairments in convective and potentially diffusive O_2_ transport. In line with this proposition, acute potentiation of cGMP signalling with phosphodiesterase 5 inhibition increases O_2_ delivery and utilisation during low-to moderate-intensity exercise in older but not young subjects (Nyberg et al., 2015b; Piil et al., 2018).

Exercise training leads to marked cardiovascular and skeletal muscle adaptations to structure and function, and physical inactivity also shares many similarities with aging when it comes to deterioration in these systems (Saltin et al., 1968; McGuire et al., 2001a, b; Hellsten and Nyberg, 2015). Hence, one obvious question is to what extent impairments in these systems are a consequence of aging, inactivity, or a combination of the two. One piece to this puzzle comes from the observation that training-induced potentiation of cGMP signaling leads to improvements in skeletal muscle blood flow and O_2_ delivery in older subjects (Piil et al., 2018). Furthermore, lifelong physical activity is associated with enhanced leg endothelial function, purinergic signalling, and ability for functional sympatholysis as well as preserved skeletal muscle oxidative metabolism during knee-extensor exercise (Mortensen et al., 2012b; Nyberg et al., 2012).

#### Cycling Exercise

The first report of an association between aging and reduced skeletal muscle blood flow was published in 1974 and was based on observations made during cycling (Wahren et al., 1974). This association has since been confirmed and in part attributed to inefficient sympatholysis (Proctor et al., 1998; Koch et al., 2003; Poole et al., 2003). During submaximal exercise, increased O_2_ extraction compensates for the lower O_2_ delivery so that leg skeletal muscle O_2_ uptake is maintained for a given absolute work rate (Proctor et al., 1998; Poole et al., 2003). Importantly, there are also reports of unaltered leg blood flow, a-vO_2_ difference, and oxidative metabolism during similar absolute submaximal work rates (Beere et al., 1999; Proctor et al., 2003), indicating that leg hemodynamics and metabolism during cycling may not solely be reflective of age *per se*.

It is well established that maximal systemic O_2_ uptake and power output during cycling declines with advancing age. The mechanisms underlying this reduction has been suggested to be lower stroke volume, heart rate, cardiac output, systemic a-vO_2_ difference, leg blood flow, and leg O_2_ delivery and utilisation, however, there is inconsistency between studies in terms of the contribution from each specific mechanism that, at least in part, may reflect differences in gender and physical activity level (Beere et al., 1999; McGuire et al., 2001a; Poole et al., 2003; Proctor et al., 2003; Proctor et al., 2004; Carrick-Ranson et al., 2020; Pandey et al., 2020).

Findings from training studies have provided some support for a more prominent role of a peripheral limitation. In one study of older men, maximal systemic O_2_ uptake was reported to increase as a result of enhanced leg blood flow and O_2_ uptake in the absence of altered central hemodynamics, highlighting an improved distribution of cardiac output to the exercising muscles in the lower extremities (Beere et al., 1999). An increase in maximal O_2_ uptake in the absence of increased cardiac output has also been reported in another study where a training-induced increase in stroke volume was counterbalanced by a decline in maximal heart rate in older men (McGuire et al., 2001b). However, lifelong endurance exercise in women was found to be associated with increased maximal systemic O_2_ uptake that was driven by enhanced stroke volume and cardiac output as a-vO_2_ difference was unaltered (Carrick-Ranson et al., 2020).

**FIGURE 3:**
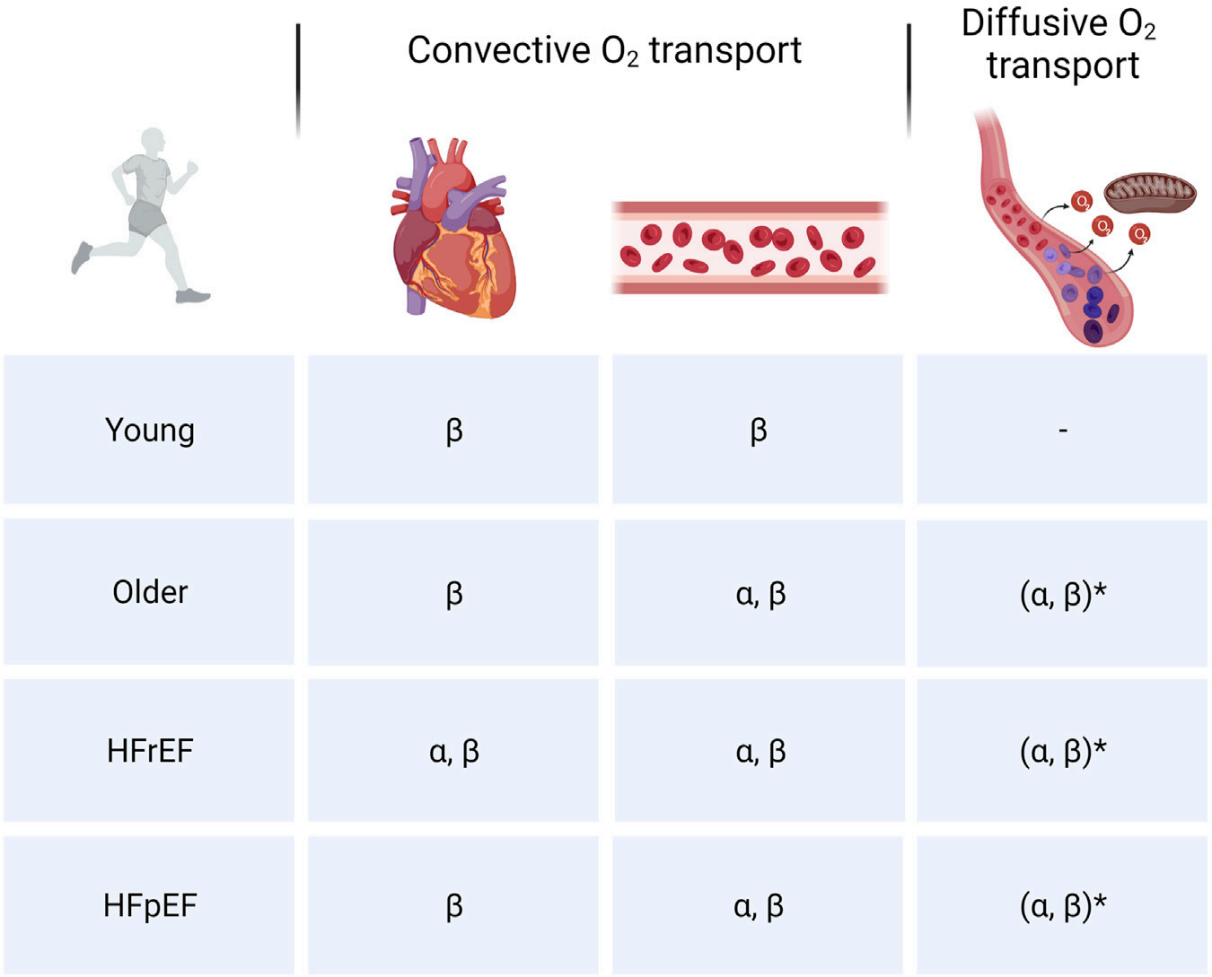
Cardiovascular limitations to skeletal muscle oxidative metabolism. Overview of limitations in convective (central and peripheral) and diffusive O_2_ transport to skeletal muscle O_2_ utilization in different populations. **α:** Intense or maximal exercise engaging a small muscle mass/submaximal exercise engaging a larger muscle mass. **β:** Intense/maximal exercise engaging a large muscle mass. *Diffusive O_2_ transport is closely linked to convective O_2_ transport. Hence, given the reductions in bulk blood flow, inherent heterogeneity of microvascular blood flow and imperfect matching between O_2_ delivery and metabolic demand at the level of the myocyte, the extent to which diffusive O_2_ transport is a limiting factor in aging and HF may be overestimated.

Based on the findings during these various types of exercise engaging the upper and lower extremities, most evidence suggest that age-related processes lead to declines in both central and peripheral components of the O_2_ transport cascade although convective and potentially diffusive O_2_ transport in skeletal muscle appear to play more prominent roles in limiting oxidative metabolism and muscle performance (Figure 3). Future studies should aim at disentangling the effects of physical inactivity and aging to further deepen our understanding of age-related declines in cardiometabolic function. Furthermore, given the underrepresentation of female subjects in studies and the previously described differences in cardiovascular adaptations to exercise training in males and females, future studies should also aim at improving our understanding of gender differences in age- and inactivity-related declines in cardiovascular structure and function.

### Heart Failure

Heart failure (HF) is defined as a clinical syndrome with symptoms and/or signs caused by a structural and/or functional cardiac abnormality that is corroborated by elevated natriuretic peptide levels and/or objective evidence of pulmonary or systemic congestion (Bozkurt et al., 2021). Common for these patients is a reduction in maximal cardiac output as well as structural and functional alterations in skeletal muscle, which at the more progressed stages of the disease has severe implications for the ability to perform even simple daily activities.

Historically, patients have been characterised and segmented by their left ventricular (LV) ejection fraction (EF) so that patients with values ≤40% were classified as HF with reduced EF (HFrEF) and patients displaying LV values ≥50% as HF with preserved EF (HFpEF). Importantly, the mechanisms underlying the cardiac insult and disease progression are distinct. In HFrEF, LV (systolic) dysfunction is driven by progressive loss of cardiomyocytes due to ischemia, infection, or toxicity whereas myocardial remodelling and LV (diastolic) dysfunction in HFpEF results from a systemic proinflammatory state induced by comorbidities such as overweight/obesity, diabetes mellitus, chronic kidney disease, and chronic obstructive pulmonary disease (Paulus and Tschope, 2013; Pandey et al., 2021). In relation to these differences in pathophysiology, there is compelling evidence that skeletal muscle, in combination with cardiac dysfunction, plays a role in the impairment of O_2_ delivery and utilisation in HFrEF (Poole et al., 2012; Hirai et al., 2015) with the peripheral component potentially being more pronounced in HFpEF (Haykowsky et al., 2015; Sarma and Levine, 2015).

HF is largely a disease of the elderly with 50% of all HF diagnoses and 90% of all HF deaths occurring in patients over the age of 70 and HFpEF has also recently been described as a geriatric syndrome (Strait and Lakatta, 2012; Pandey et al., 2021). Although aging does not cause HF, the structural and functional changes associated with aging does lower the threshold for manifestations of the disease as many of these are integral components of HF pathology. However, to what extent HF-related impairments in O_2_ transport are a direct effect of age remain to be established. The following sections are meant to highlight the current understanding of mechanisms operating in HF.

#### HFrEF

In addition to the apparent central limitation in HFrEF, peripheral alterations may compromise the matching of O_2_ delivery and demand. These include, albeit that a great degree of heterogeneity exists in the patient population, a shift in fibre type distribution from slow-twitch oxidative to fast-twitch glycolytic fibres, fibre atrophy, reduced mitochondrial volume density, loss of capillaries supporting red blood cell flux, and impaired vascular function (Drexler et al., 1992; Poole et al., 2012; Poole et al., 2021). These structural and functional alterations can all have a substantial impact on skeletal muscle convective and diffusive O_2_ delivery capacity (Poole et al., 2012).

##### Handgrip Exercise

If both the capacity to increase convective and diffusive O_2_ transport are attenuated in response to physical activity, skeletal muscle O_2_ utilisation could potentially be impaired even during small muscle mass exercise. During sustained handgrip exercise, blood flow (Wiener et al., 1986; Massie et al., 1987; Arnold et al., 1990; Shoemaker et al., 1999) and O_2_ uptake (Arnold et al., 1990; Shoemaker et al., 1999) have been reported to be similar between HFrEF patients and controls at submaximal work rates. However, a steeper slope of the Pi/PCr-to-power output relationship, lower muscle and venous pH, and higher venous H^+^ were detected in patients (Wiener et al., 1986; Massie et al., 1987; Shoemaker et al., 1999), suggesting an altered skeletal muscle metabolism that is in part driven by augmented glycolytic flux. These observations also indicate that skeletal muscle hemodynamics are not altered when exercise is confined to forearm muscles but does not exclude the possibility that O_2_ delivery to muscles in the upper extremities may be compromised during conditions where a substantial demand for cardiac output and increase in sympathetic drive are prominent.

##### Knee-Extensor Exercise

When knee-extensor exercise was performed with one leg, skeletal muscle blood flow and O_2_ uptake were found to be similar between HFrEF patients and controls at any given submaximal absolute work rate (Magnusson et al., 1997; Munch et al., 2018). When performing knee-extensor exercise with both legs, lower cardiac output and skeletal muscle perfusion were observed in patients, while control subjects maintained perfusion of the exercising muscles that was enabled *via* higher cardiac output. At peak intensity, blood flow and O_2_ uptake were similar in both legs of the controls while these variables were reduced in patients. Despite apparent impairments in O_2_ delivery in HFrEF, the a-vO_2_ difference was not different between the two groups at peak exercise with one and two legs (Magnusson et al., 1997). Similar findings were made in two separate studies in which skeletal muscle O_2_ delivery, a-vO_2_ difference, and O_2_ uptake were found to be comparable in HFrEF patients and controls during submaximal knee-extensor exercise performed at similar absolute work rates (Esposito et al., 2015), whereas O_2_ supply and utilisation, but not a-vO_2_ difference, were lower at peak exercise in HFrEF patients (Esposito et al., 2011). Moreover, muscle O_2_ diffusive capacity was lower in patients and in that same study the increase in maximal knee-extensor O_2_ uptake was also shown to be the result of a training-induced increase in both convective and diffusive O_2_ transport capacity (Esposito et al., 2011). Regarding skeletal muscle diffusive O_2_ capacity, it should be mentioned that this variable is portrayed as the ratio of peak skeletal muscle O_2_ consumption and O_2_ pressure gradient between microvessels and mitochondria. However, in practice it is calculated as the ratio of blood flow x a-vO_2_ difference and a-vO_2_ pressure gradient across the entire muscle/limb. Hence, reports of reduced skeletal muscle diffusive O_2_ capacity may to a large extent reflect impairments in convective O_2_ transport and blood flow distribution as these variables are severely affected in HFrEF.

Reduced blood flow during single-leg submaximal knee-extensor exercise has also been reported in HFrEF patients but is unclear how this affected oxidative metabolism as sampling of arterial and venous blood was not performed (Barrett-O'Keefe et al., 2014). Taken collectively, these findings provide evidence that limitations in both skeletal muscle convective and potentially diffusive O_2_ transport contribute to reduced peak O_2_ uptake in HFrEF patients during knee-extensor exercise, which contrasts with healthy individuals where the site of limitation resides at the level of the mitochondria.

##### Cycling Exercise

Central hemodynamics, leg blood flow, and metabolic responses to upright cycle exercise have also been assessed in HFrEF patients. At similar absolute work rates, cardiac output, leg blood flow, and leg vascular conductance were found to be lower at all submaximal and maximal intensities in HFrEF patients compared to controls (Sullivan et al., 1989). This impairment in skeletal muscle perfusion was associated with an increased a-vO_2_ difference, however, O_2_ uptake was only preserved at the lowest intensity with O_2_ utilisation falling when the work rate was increased. The higher vascular resistance in these patients was an important contributor to systemic vascular resistance that allowed preservation of mean arterial pressure and the maintenance of sufficient perfusion pressure across vital organs at all work rates. In line with this observation, peak systemic and leg O_2_ delivery, a-vO_2_ difference, muscle O_2_ diffusional conductance, and O_2_ uptake was reported to be lower in HFrEF patients (Esposito et al., 2011; Dhakal et al., 2015).

To disclose potential peripheral limitations to O_2_ uptake in HFrEF, insights from studies using training with a small muscle group can be informative as such protocols limits cardiac adaptations. By using this approach, increases in capillary-to-fibre ratio, mitochondrial volume density, oxidative enzyme activity, type 1 fibre fraction, and functional sympatholysis have been reported alongside higher levels of peak O_2_ delivery, a-vO_2_ difference, muscle O_2_ diffusional conductance, and O_2_ uptake during knee-extensor exercise (Magnusson et al., 1996; Tyni-Lenne et al., 1999; Esposito et al., 2011; Munch et al., 2018). Importantly, these peripheral adaptations were also associated with increases in peak systemic O_2_ uptake during cycling exercise (Tyni-Lenne et al., 2001; Esposito et al., 2011). Furthermore, cycle exercise training increased peak leg blood flow, O_2_ delivery, a-vO_2_ difference, and O_2_ uptake whereas the increase in cardiac output did not reach statistical significance (Sullivan et al., 1988). Hence, reductions in both cardiac output and skeletal muscle convective and diffusive O_2_ transport capacity in skeletal muscle appear to contribute to impaired peripheral oxidative capacity and exercise intolerance in HFrEF patients. This peripheral limitation during exercise engaging both legs is in line with that observed during knee-extensor exercise and should be viewed in the context of augmented peripheral vascular resistance and insufficient muscle perfusion needed to uphold mean arterial pressure.

#### HFpEF

HFpEF, which is characterized by abnormal relaxation of the LV and decreased LV compliance, is a multiorgan geriatric syndrome driven by mechanisms related to multimorbidity, systemic inflammation, obesity, aging, and physical inactivity (Paulus and Tschope, 2013; Pandey et al., 2021). Hence, HFpEF is distinct from HFrEF in many regards as also evidenced by findings from clinical trials in which neurohumoral blockade improves mortality and HF hospitalisations in HFrEF but not HFpEF. Recently, the angiotensin receptor-neprilysin inhibitor sacubitril-valsartan and the sodium–glucose cotransporter 2 inhibitor empagliflozin were shown to also have clinical benefits in HFpEF; however, an attenuation of benefits in patients with higher EF was evident in both trials (Solomon et al., 2019; Anker et al., 2021).

Following the onset of exercise, HFpEF patients display greater increases in pulmonary arterial pressure, pulmonary capillary wedge pressure, LV end diastolic pressure, and late systolic load that in conjunction with a lower increase in heart rate results in reduced cardiac output (Borlaug et al., 2010; Pandey et al., 2021). Although exercise-induced pulmonary oedema is a primary cause of exertional intolerance in HFpEF, abnormalities in skeletal muscle O_2_ uptake are apparent even during submaximal exercise (Sarma and Levine, 2015). Contributors to this peripheral maladaptation to acute exercise in HFpEF include reduced vascular function, capillary-to-fibre ratio, mitochondrial content and oxidative capacity, loss of type 1 fibres, and muscle fat infiltration that all may affect skeletal muscle convective and diffusive O_2_ transport and utilisation (Kitzman et al., 2014; Sarma and Levine, 2015; Marechaux et al., 2016; Molina et al., 2016; Pandey et al., 2021).

##### Handgrip and Knee-Extensor Exercise

Only very few studies have investigated hemodynamic and metabolic variables in HFpEF patients during isolated forearm and knee-extensor exercise. In one study, skeletal muscle vascular conductance and blood flow were found to be reduced at higher submaximal intensities in patients in the absence of macrovascular dysfunction and dysregulated central hemodynamics (Ratchford et al., 2020). In another study, a-vO_2_ difference was reduced across all brachial blood flows in patients, however, peak O_2_ uptake was preserved in HFpEF through a statistically non-significant enhancement (∼14%) of blood flow (Zamani et al., 2020). Notably, forearm diffusional O_2_ conductance was not different among HFpEF patients and healthy control subjects. During knee-extensor exercise, HFpEF patients have been shown to exhibit marked exercise intolerance compared to controls, with almost 25% of patients unable to continue beyond unloaded ergometer exercise (Lee et al., 2016). These patients also displayed lower skeletal muscle vascular conductance and blood flow during exercise that was not related to dysregulated central hemodynamics. These initial observations during small muscle mass exercise are indicative of abnormalities in skeletal muscle convective O_2_ transport but additional studies are warranted to confirm this.

##### Cycling and Running

By using a modified Astrand–Saltin incremental treadmill protocol, it was demonstrated that HFpEF patients were able to increase cardiac output and other indices of cardiac reserve to a similar extent as that of controls during submaximal and maximal running (Bhella et al., 2011). This preservation of central hemodynamics was, however, associated with lower systemic a-vO_2_ difference and O_2_ uptake, which was likely to be caused by a lower capacity for skeletal muscle oxidative metabolism as measured by magnetic resonance spectroscopy. Interestingly, a similar hyperdynamic response (∆cardiac output/∆O_2_ uptake slope) is observed in patients with mitochondrial myopathies (Taivassalo et al., 2003). In line with this peripheral limitation, impaired skeletal muscle O_2_ extraction attributable to impaired diffusive O_2_ transport and utilization has also been shown in HFpEF patients during upright maximal intensity cycling (Dhakal et al., 2015; Houstis et al., 2018). These studies also demonstrated lower cardiac output and systemic O_2_ uptake and it is important to note that the vast majority (97%) of patients with HFpEF harboured defects at multiple steps of the O_2_ pathway with a high degree of heterogeneity in terms of identity and magnitude (Houstis et al., 2018). This heterogeneity resonates well with a multiorgan syndrome driven by a broad range of pathophysiological mechanisms and underscores that the extent to which a disease-induced alteration in convective and diffusive O_2_ transport and/or utilization may contribute to impaired skeletal muscle metabolic function will be patient and context-dependent.

Several clinical trials have evaluated the efficacy of exercise training in improving systemic O_2_ uptake and cardiac function and found that peak O_2_ uptake increases without significant changes in LV systolic or diastolic function measured at rest (Pandey et al., 2015). Evidently, more training studies are needed to assess whether central hemodynamics are altered during exercise and to what extent skeletal muscle convective and diffusive O_2_ transport and utilization are altered with training to shed more light on central *vs.* peripheral limitations in HFpEF.
